# A Novel Prognostic Nomogram for Gallbladder Cancer after Surgical Resection: A Single-Center Experience

**DOI:** 10.1155/2021/6619149

**Published:** 2021-02-08

**Authors:** Zuyi Ma, Fengying Dong, Zhenchong Li, Zehao Zheng, Zixuan Zhou, Hongkai Zhuang, Chunsheng Liu, Bowen Huang, Shanzhou Huang, Yiping Zou, LinLing Yang, Yuanfeng Gong, Chuanzhao Zhang, Baohua Hou

**Affiliations:** ^1^Shantou University of Medical College, Shantou 515000, China; ^2^Department of General Surgery, Guangdong Provincial People's Hospital, School of Medicine, South China University of Technology, Guangzhou 510080, China; ^3^Forth Department of Geriatrics, General Hospital of Southern Theater Command, PLA, Guangzhou 510080, China; ^4^Department of General Surgery, Peking Union Medical College Hospital, Chinese Academy of Medical Sciences and Peking Union Medical College, Beijing 100730, China; ^5^Guangzhou Medical University, Guangzhou 511436, China; ^6^Department of Hepatobiliary Surgery, The Affiliated Cancer Hospital and Institute of Guangzhou Medical University, Guangzhou 510095, China

## Abstract

**Background:**

Gallbladder cancer (GBC), which accounts for more than 80% of biliary tract malignancies, has a poor prognosis with an overall 5-year survival less than 10%. The study aimed to identify risk factors and develop a predictive model for GBC following surgical resection.

**Methods:**

98 GBC patients who underwent surgical resection from Guangdong Provincial People's Hospital were enrolled in the study. Cox-regression analysis was performed to identify significant prognostic factors. A nomogram was constructed and Harrell's concordance index, calibration plot, and decision cure analysis were used to evaluate the discrimination and calibration of the nomogram.

**Results:**

Liver resection, tumor size, perineural invasion, surgical margin, and liver invasion were identified as independent risk factors for overall survival (OS) in GBC patients who underwent surgical resection. Based on the selected risk factors, a novel nomogram was constructed. The C-index of the nomogram was 0.777, which was higher than the American Joint Committee on Cancer (AJCC) staging system (0.724) and Nevin staging system (0.659). Decision cure analysis revealed that the nomogram had a better net benefit and the calibration curves for the 1-, 3-, and 5-year survival probabilities were also well matched with the actual survival rates. Lastly, high-risk GBC were stratified based on the scores of the nomogram and we found high-risk GBC were associated with both worse OS and disease-free survival (DFS).

**Conclusion:**

We developed a nomogram showing a better predictive capacity for patients' survival of resected GBC than the AJCC staging systems. The established model may help to stratify high-risk GBC and facilitate decision-making in the clinic.

## 1. Introduction

Gallbladder cancer (GBC) is relatively uncommon with an overall incidence of 2.3 per 100,000 people, but it accounts for more than 80% of biliary tract malignancies [1]. Patients with GBC have a poor prognosis with an overall 5-year survival less than 10% [2]. Some studies have suggested that elevated carbohydrate antigen 19-9 (CA19-9) and carcinoembryonic antigen (CEA) were associated with the poor prognosis of GBC [3, 4]. Other clinicopathological characters including positive surgical margin, lympho-vascular invasion, perineural invasion, and liver invasion were also considered as the risk factors for GBC patients prognosis [5–7]. Nevin staging system was simple and convenient for surgeons to evaluate the GBC patients and approximately predicted the GBC prognosis [8]. Regarding traditional tumor stages, tumor-node-metastasis (TNM) staging from the American Joint Committee on Cancer (AJCC) has been considered as the most valuable predictor of GBC prognosis [9]. However, the staging systems mainly focus on pathological outcomes but ignore some demographic characteristics.

Recently, investigators tried to develop novel prognostic models for patients with GBC based on different cohorts [10, 11]. Wang et al. established a nomogram to make individualized estimates of survival by using the Surveillance, Epidemiology, and End Results (SEER) Medicare database in 2011 [11]. They predicted that GBC patients with at least T2 or N1 disease would gain a survival benefit from adjuvant chemoradiotherapy. However, due to lack of much clinical information and various pathological factors, using SEER Medicare database for prediction model establishment could cause much bias.

In the current study, we identified prognostic factors from comprehensive tumor characteristics and surgical parameters based on the data of our institution. Furthermore, we developed a novel nomogram to predict the prognosis of patients with GBC after surgical resection.

## 2. Methods

### 2.1. Data Collection

From January 1st, 2008, to January 1, 2019, data of patients who underwent cholecystectomy with or without partial hepatectomy and with pathologically confirmed GBC at Guangdong Provincial People's Hospital were reviewed and included in the present study. The inclusion criteria were as follows: (1) GBC was primarily diagnosed; (2) had undergone cholecystectomy with or without wedge liver resection and with pathologically confirmed GBC; (3) American Society of Anesthesiologists (ASA) Score < III; (4) no neoadjuvant treatments before surgery. Patients who (1) had incomplete clinical and pathological data, (2) had other malignancy, (3) had distant metastasis, and (4) died within 30 days after surgery were excluded from this study. Patients' demographic, clinical, and pathological information and corresponding variables were manually collected, including age, sex, body mass index (BMI), diabetes mellitus (DM), jaundice, gallbladder stone, serum albumin, CA19-9, carbohydrate antigen 125 (CA125), CEA, tumor size, surgical margin, liver resection, lymph node resection, tumor grade, lymphovascular invasion, perineural invasion, liver invasion, and lymph node positive rate (LNR). GBC stage and postoperative pathologic TNM (pTNM) information was determined by using the AJCC 8th edition (AJCC-8) classification system [9].

Different surgeries were performed for patients based on the TNM stage in our center: for patients at Tis or T1a stage, simple cholecystectomy was performed; for patients from T1b to T3 stage, not only cholecystectomy but also the wedge resection and lymph node dissection were performed to obtain the radical resection; for T4 stage, extended resection including peripheral organ resection was performed for getting a better prognosis. The primary end points were overall survival (OS) and disease-free survival (DFS), which were defined as the date of surgery to the death and recurrence or last follow-up, respectively. This study was approved by the Institutional Ethics Committee of the Guangdong Provincial People's Hospital.

### 2.2. Statistical Analysis and Nomogram Construction

Statistical analyses were performed by SPSS 20.0 and *R* version 4.0.0 software (http://www.r-project.org/). *P*-value <0.05 was regarded as statistically significant and all tests were two-sided. Cutoff values for tumor size, CA19-9, CA12-5, CEA were all defined by the receiver operating characteristic curve (ROC) analyses with the help of SPSS 20.0. Cox proportional hazards regression was used to evaluate potential risk factors of prognosis. With the help of *rms* package of *R*, the visual nomogram based on the potential risk factors was constructed. The discrimination performance of the nomogram was assessed by using the concordance index (C-index). Overfit bias was decreased by bootstrap validation including 1,000 resamples. The calibration of the nomogram was estimated by using a calibration plot. Decision curve analysis (DCA) was conducted to assess the clinical performance and the net benefit of the nomogram. Patients were divided into a high-risk group and a low-risk group using the median cutoff of the risk score. Then, the log-rank tests and Kaplan–Meier analyses were performed using the survival *R* package between the high-risk and low-risk group to assess the predictive ability of the prognostic nomogram.

## 3. Results

### 3.1. Patients Characteristics and Follow-Up

Based on the criteria, 98 eligible patients were finally enrolled into our study. Patients' clinicopathologic characteristics above are listed in Table 1. Based on the best cutoff values, tumor size, carbohydrate antigen 19-9, carbohydrate antigen 12-5, and carcinoembryonic antigen were divided into two groups and were set as cCA-19-9, cCA12-5, cCEA, and ctumor size. The median follow-up time was 28.5 months (range, 12–126 months). Overall, the median OS and DFS were 20 months and 14.5 months. The 1-, 3-, and 5-year OS and DFS rates were 73.3%, 48.8%, and 19.2% and 65.5%, 46.83%, and 14.7%, respectively.

### 3.2. Identification of Independent Prognostic Factors for GBC

**Figure 1:**
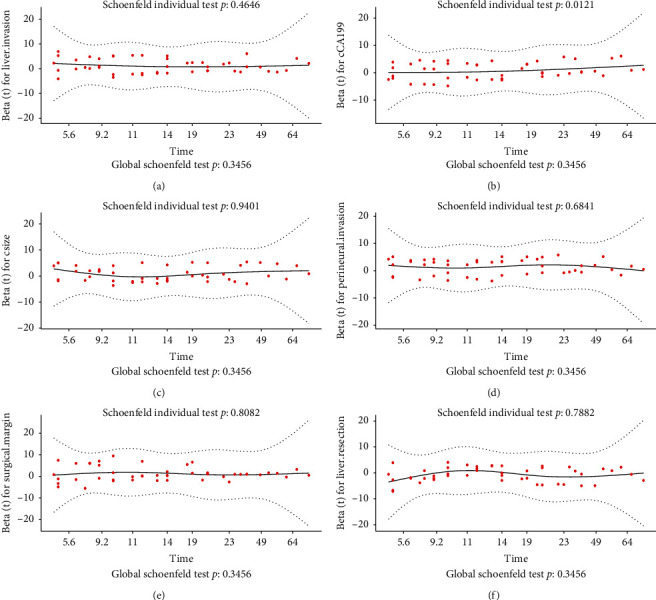
Schoenfeld residuals analysis of liver invasion (a), cCA19-9 (b), ctumor size (c), perineural invasion (d), surgical margin (e), and liver resection (f).

Cox regression analysis was performed to identify the prognostic factors for GBC. After performing the univariate cox regression analysis, jaundice, tumor grade, lymphovascular invasion, perineural invasion, surgical margin, liver invasion, cCA-19-9, cCA12-5, cCEA, and ctumor size were regarded as potential risk factors. All significant univariable predictors and some other important clinical variables (e.g., liver resection) were enrolled into multivariate cox regression analysis (Table 2). Finally, the results indicated that perineural invasion, surgical margin, liver invasion, liver resection, cCA-19-9, and ctumor size were the independent prognostic factors for OS in patients with GBC who underwent the surgical therapy (Table 2). Further, we performed the Schoenfeld residuals analysis to evaluate whether the proportional hazards assumption was valid. We show in Figure 1 that the *P*-values for liver resection (Figure 1(a)), cCA19-9 (Figure 1(b)), ctumor size (Figure 1(c)), perineural invasion (Figure 1(d)), surgical margin (Figure 1(e)), and liver invasion (Figure 1(f)) are 0.4646, 0.0121, 0.9401, 0.6841, 0.8082, and 0.7882 separately and the *P*-value for global test is 0.3456. These results indicated that liver resection, ctumor size, perineural invasion, surgical margin, and liver invasion were satisfied for the proportional hazards assumption. However, cCA19-9 was unsatisfied and excluded in the multivariate model.

### 3.3. Construction and Validation of a Novel Prognostic Nomogram for OS

**Figure 2:**
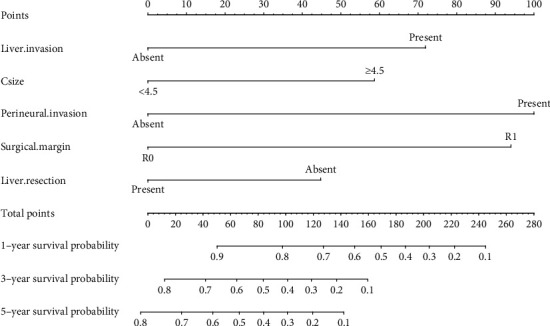
The nomogram for predicting the 1-, 3-, and 5-year survival of gallbladder cancer patients.

**Figure 3:**
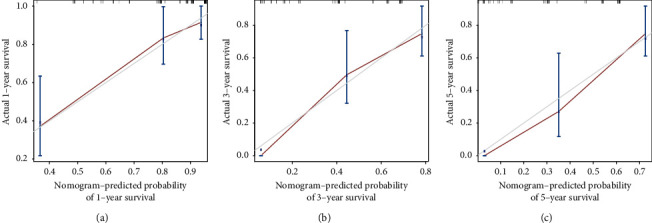
Calibration plots of the nomogram for 1-year (a), 3-year (b), and 5-year (c) survival prediction of gallbladder cancer patients.

**Figure 4:**
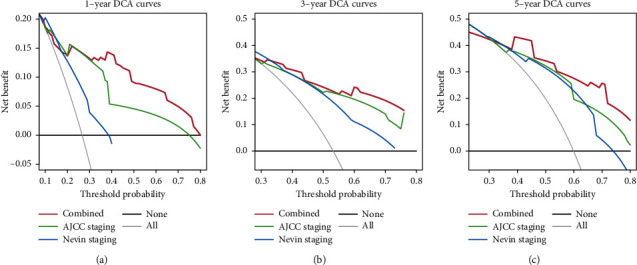
Decision curve analysis and Kaplan–Meier analysis of the nomogram. (A-C) Decision curve analysis of the nomogram for 1-year (a), 3-year (b), and 5-year (c) survival prediction of gallbladder cancer patients.

Based on the results of multivariate cox regression analysis, perineural invasion, surgical margin, liver invasion, liver resection, and ctumor size were selected as the significant factors and were integrated to establish a nomogram for predicting the OS of GBC (Figure 2). After applying the bootstrap validation, the bias-corrected C-index of the nomogram was 0.777, which was higher than the AJCC staging system (0.724) and Nevin staging system (0.659). In our OS nomogram, perineural invasion was given 100 points while R0 surgical margin was assigned 94.0578 points. The presence of liver invasion was assigned as 71.90517 points, while conducting the liver resection treatment would be given 44.78229 points. A tumor size less than 4.5 cm was scored as 0, and a tumor size ≥4.5 cm was scored as 58.71448 points. Finally, the total points can be calculated and converted to obtain the probability of 1-, 3- and 5-year overall survival. The calibration curves for the 1-year (Figure 3(a)), 3-year (Figure 3(b)), and 5-year (Figure 3(c)) survival probabilities were also well matched with the actual survival rates, which showed that the nomogram could predict the 1-, 3-, and 5-year OS in our cohort accurately. DCA were conducted to assess the clinical performance and the net benefit of the nomogram. It is revealed that the nomogram had a better net benefit with a wider range of threshold probabilities than AJCC staging system and Nevin stagging system for both 1-year (Figure 4(a)), 3-year (Figure 4(b)), and 5-year (Figure 4(c)) OS, which could provide a better clinical benefit for the clinicians and patients.

### 3.4. The Association between High-Risk GBC and Patients' Survival

**Figure 5:**
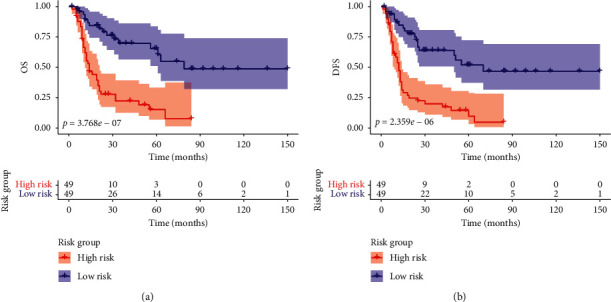
Kaplan–Meier analysis of gallbladder cancer patients stratified by median risk score. Patients in the high-risk group had a significantly shorter overall survival (a) and disease-free survival (b) than those in the low-risk group.

The cohort was divided into high-risk group and low-risk group based on the median of the risk score and Kaplan–Meier analysis was performed to evaluate patients' OS and DFS in the two groups. The results showed that patients in high-risk group had both shorter OS (*P*-value = 3.768*e* − 07) and DFS (*P*-value = 2.359*e* − 06) than those in low-risk group (Figures 5(a) and 5(b)), indicating a significant unfavorable outcome for high-risk GBC.

## 4. Discussion

Despite continuous advances in the diagnosis and management of GBC in recent years, no major breakthrough for effective biomarkers nor treatment strategies have emerged. Surgical resection remains the only potentially curative treatment for patients with GBC [12]. However, the prognosis of GBC patient was still very poor with a 5-year survival rate of only about 10% [13]. For patients who underwent the surgical resection, it is of great interest to develop an accurate prognostic model and identify the high-risk patients.

In the current study, perineural invasion, surgical margin, liver invasion, liver resection, and ctumor size were selected as the significant predictive factors and integrated to establish a nomogram for predicting the OS of GBC. The C-index of the nomogram was 0.775, which was higher than the C-index for the 8th edition TNM stage system (0.724). The calibration plot revealed a good coincidence between predicted survival rate and actual survival rate and decision cure analysis also demonstrated that our nomogram had a strong potential clinical application. The Kaplan–Meier plot suggested that patients in high-risk group had significantly poorer recurrence-free survival and overall survival than those in low-risk group. The results above indicated that our nomogram had outstanding consistency, calibration, discrimination, and stratification for prediction of GBC patients who underwent surgical resection.

Perineural invasion is relatively common in GBC patients at T2 and T3 stage [14]. Some studies had reported that perineural invasion was significantly related to residual disease in incidental gallbladder carcinoma. Other researches also indicated that perineural invasion was associated with poor prognosis of GBC patients after surgical resection [15, 16]. Perineural invasion was almost not observed in patients with stage T1 cancer, while it was detected rarely in distal-type tumors, which would develop various types of recurrences [15]. A current study indicated that an increasing affinity for nerve of tumor cells may be caused by a reciprocal interaction between the cancer cells and the microenvironment of the host nerve [17]. Interestingly, many researches had indicated that tumor size may not be the independent risk factor for the prognosis of the GBC patients [10, 18]. However, after transforming the tumor size into a categorical variable, we found that GBC patients with tumor sizes <4.5 cm may have better prognoses.

A small and single-center retrospective study suggested that liver invasion may be the only independent risk factor of GBC even at a very early stage [19]. In our study, we confirmed that liver invasion affected significantly on the prognosis of GBC patients treated after surgery. Liver resection is an essential part of the radical surgery for the GBC patients at T1b or higher stage. There were two alternative hepatectomy choices for GBC, which were hepatic wedge resection around the gallbladder fossa or hepatic IVb/V segmentectomy. The purpose of the former operation was to obtain a negative resection margin, while the latter operation was anatomic resection to obtain an additional oncologic benefit [20]. Recently, a multicenter study showed that T2 GBC patients who underwent the liver resection had better five-year survival rate compared to those who received no liver resection [21]. Moreover, their results indicated patients who underwent hepatic wedge resection or IVb/V segmentectomy had similar disease-free survival. Other two multicenter studies also reported that there was no difference in survival rate or recurrence rates between the groups that underwent hepatic wedge resection and IVb/V segmentectomy [22, 23]. In our center, most of GBC patients received the hepatic wedge resection and achieved negative resection margin. Large multicenter randomized control trials (RCTs) or matched studies regarding the safety and effectiveness of hepatic wedge resection versus IVb/V segmentectomy in GBC are needed in the future.

There are some limitations in our study. First, our nomogram may not be suitable for all GBC patients. Only patients without distant metastasis and who received surgical resection fit the model. Besides, the study was based on data from a single institute with a relatively small sample size. Independent cohorts from other centers are needed for future validation studies.

In conclusion, liver resection, tumor size, perineural invasion, surgical margin, and liver invasion played crucial roles in the prognosis of GBC patients. Based on these clinicopathological risk factors, we developed and validated a novel nomogram to predict the overall survival for GBC patients following surgical resection, which may facilitate decision-making in the clinic.

## Figures and Tables

**Table 1:** Clinicopathological characteristics of patients.

Patient characteristics		Patient number (*n* = 98)
Age (years)	≥60	55
	＜60	43

Gender	Male	42
	Female	56

BMI (kg/㎡)	≥24	20
	＜24	78

Jaundice	Yes	20
	No	78

Diabetes mellitus	Yes	15
	No	83

Gallbladder stone	Yes	46
	No	52

T stage	Tis/T1	12
	T2	54
	T3	24
	T4	8

AJCC stage	I ∼ IIIA	67
	IIIB ∼ IVA	31

Tumor grade	Low	29
	Medium	61
	High	13

Lymphovascular invasion	Yes	20
	No	78

Perineural invasion	Yes	34
	No	64

Surgical margin	R0	80
	R1	18

Liver invasion	Yes	23
	No	75

Lymph node positive rate (LNR)	≥0.28	24
	＜0.28	74

Liver resection	Yes	66
	No	32

Lymph node dissection (LND)	Yes	55
	No	43

Tumor size (cm)	≥4.5	26
	＜4.5	72

CA125 (U/ml)	≥12	62
	＜12	36

CA19-9 (U/ml)	≥58.5	32
	＜58.5	66

CEA (ng/ml)	≥5	22
	＜5	76

BMI, body mass index; CA125, carbohydrate antigen 125; CA19-9, carbohydrate antigen 19-9; CEA, carcinoembryonic antigen; the American Joint Committee on Cancer (AJCC) stage is according to the AJCC 8th edition.

**Table 2:** Univariate and multivariate Cox regression analyses for survival.

Patient characteristics		Univariate analysis		Multivariate analysis	
		HR (95%CI)	*P*-value	HR (95%CI)	*P*-value
Age	<60	1			
	≥60	1.484(0.826∼2.666)	0.187		

Gender	Male	1			
	Female	1.559(0.834∼2.917)	0.164		

BMI	<24	1			
	≥24	0.585(0.248∼1.379)	0.22		

Jaundice	Absent	1		1	
	Present	2.182(1.105∼4.308)	**<0.05**	**1.407(0.624∼3.175)**	0.411

Diabetes mellitus	Absent	1			
	Present	0.917(0.362∼2.319)	0.855		

Gallbladder stone	Absent	1			
	Present	1.199(0.676∼2.127)	0.534		

AJCC stage	I ∼ IIIA	1			
	IIIB-IVB	**3.66(1.963∼6.825)**	**<0.05**	1.269(0.422∼3.811)	0.671

Tumor grade	High	1			
	Medium	7.049(0.952∼52.170)	0.056	5.707(0.731∼44.527)	0.10
	Low	**11.186(1.470∼85.170)**	**0.020**	6.340(0.702∼57.291)	0.10

Lymphovascular invasion	Absent	1		1	
	Present	1.877(0.930∼3.787)	0.0788	1.4160(0.635∼3.156)	0.395

Perineural invasion	Absent	1		1	
	Present	**3.949(2.189∼7.122)**	**<0.05**	**3.411(1.671∼6.963)**	**<0.05**

Surgical margin	R0	1		1	
	R1	**4.234(2.028∼8.839)**	**<0.05**	**3.102(1.201∼8.013)**	**<0.05**

Liver invasion	Absent	1		1	
	Present	**3.026(1.602∼5.718)**	**<0.05**	**3.00(1.336∼6.736)**	**<0.05**

Lymph node positive rate	<0.28	1		1	
	≥0.28	**3.007(1.502∼6.020)**	**<0.05**	1.019(0.319∼3.251)	0.975

Liver resection	Absent			1	
	Present	0.771(0.426∼1.395)	0.39	**0.3593(0.162∼0.797)**	**<0.05**

Tumor size	<4.5	1		1	
	≥4.5	1.807(0.9715∼3.362)	0.061	**2.660(1.248∼5.672)**	**<0.05**

CA125	<12	1		1	
	≥12	**2.639(1.338∼5.025)**	**<0.05**	**1.663(0.760∼3.643)**	**0.203**

CA19-9	<58.5	1		1	
	≥58.5	**3.539(1.936∼6.468)**	**<0.05**	**2.254(1.070∼4.745)**	**<0.05**

CEA	<5	1		1	
	≥5	**3.155(1.622∼6.136)**	**<0.05**	0.558(0.237∼1.314)	0.182

BMI, body mass index; CA125, carbohydrate antigen 125; CA19-9, carbohydrate antigen 19-9; CEA, carcinoembryonic antigen; the American Joint Committee on Cancer (AJCC) stage is according to the AJCC 8th edition.

## Data Availability

The underlying data supporting the results of our study are provided in the article.

## Abbreviations

GBC: Gallbladder cancer
TNM: Tumor-node-metastasis
AJCC: American Joint Committee on Cancer
SEER: Surveillance, Epidemiology, and End Results
CA19-9: Carbohydrate antigen 19-9
CA125: Carbohydrate antigen 125
CEA: Carcinoembryonic antigen
LNR: Lymph node positive rate
OS: Overall survival
DFS: Disease-free survival
ROC: Receiver operating characteristic curve
DCA: Decision curve analysis.
